# Maternal Anemia and Low Birth Weight: A Systematic Review and Meta-Analysis

**DOI:** 10.3390/nu10050601

**Published:** 2018-05-12

**Authors:** Ana C. M. G. Figueiredo, Isaac S. Gomes-Filho, Roberta B. Silva, Priscilla P. S. Pereira, Fabiana A. F. Da Mata, Amanda O. Lyrio, Elivan S. Souza, Simone S. Cruz, Mauricio G. Pereira

**Affiliations:** 1 Faculty of Health Sciences, University of Brasilia, Brasília 70910-900, Distrito Federal, Brazil; roberta.silva.borges@gmail.com (R.B.S.); priperez83@gmail.com (P.P.S.P.); mauriciogpereira@gmail.com (M.G.P.); 2 Department of Health, Feira de Santana State University, Feira de Santana 44036-900, Bahia, Brazil; isuzart@gmail.com; 3 Faculty of Medical Sciences, University of Brasilia; Brasília 70910-900, Distrito Federal, Brazil; fagfigueiredo@hotmail.com; 4 Department of Epidemiology, Federal University of Recôncavo da Bahia, Santo Antônio de Jesus 44.570-000, Bahia, Brazil; amandalyryo@hotmail.com (A.O.L.); elivan-silva@outlook.com (E.S.S.); simone.seixas1@gmail.com (S.S.C.)

**Keywords:** anemia, pregnancy, low birth weight, cohort study, systematic review

## Abstract

Objective: To systematically analyze the relationship between maternal anemia and low birth weight. Methods: A search of studies was conducted in the main databases (Medline, Embase, Scopus, Web of Science, SciELO, and Lilacs), the gray literature, and the reference lists of selected articles. Cohort and case-control studies that met the eligibility criteria were included in the review. There was no limitation on the language or date of publication. Article selection and data extraction were performed by two independent reviewers. Meta-analyses with random effects, subgroup analyses and meta-regressions were performed. Publication bias was measured using Egger regression and visual funnel plot inspection. Results: A total of 7243 articles were found, of which 71 comprised the systematic review and 68 were included in the meta-analyses. Maternal anemia was associated with low birth weight with an adjusted OR: 1.23 (95% CI: 1.06–1.43) and I^2^: 58%. The meta-regressions confirmed that the sample size and the methodological quality may partially explain the statistical heterogeneity. Conclusions: Maternal anemia was considered a risk factor for low birth weight.

## 1. Introduction

Worldwide, approximately 7–15% of all live births each year are of low birth weight, a gestational outcome that is considered a major public health problem and is more prevalent in countries with fewer financial resources [1].

Children born weighing less than 2500 g are more prone to infant morbidity and mortality [2,3]. Inadequate biological, social, economic, environmental, and lifestyle factors, either prior to or during pregnancy, may favor low birth weight [2,4,5]. Some nutritional aspects, such as a low nutritional diet and inadequate weight gain during pregnancy, contribute to a lower intake of the nutrients considered important for fetal growth, such as B vitamins and iron [6].

Ionic iron is the mineral that promotes the formation of new hemoglobin and is the main source of energy and oxygen transportation to the organs of the body [6]. Maternal anemia can develop due to both the unavailability of this element in the extracellular environment for erythropoiesis and the presence of infectious processes, which may influence the metabolism of new hemoglobin [7]. In general, the diagnosis of maternal anemia is defined by hemoglobin levels below 11 g/dL [8,9,10].

Reduced levels of hemoglobin favor changes in placental angiogenesis, limiting the availability of oxygen to the fetus and, consequently causing potential restriction of intrauterine growth and low birth weight [11]. Pregnant women with hemoglobin levels below 11 g/dL are at higher risk of having low birth weight children compared with women who do not have anemia during pregnancy [12]. 

The most recent data for overall prevalence of maternal anemia, estimated in 2011, was 38.2%. The event occurs throughout the world, and only in North America is its prevalence less than 20%. The prevalence of maternal anemia is distributed among the continents as follows: Europe (24.5%), Latin America and the Caribbean (28.3%), Oceania (29%), Asia (39.3%), and Africa (44.6%) [8]. Due to the worldwide occurrence of this disease, maternal anemia demands attention, not only because it affects the health condition of the mother, but also because it is related to undesirable gestational outcomes [8]. 

Despite the need to investigate maternal anemia and low birth weight, two relevant public health problems, there are few robust reviews which include women from diverse countries and socioeconomic conditions. Only one systematic review was found and it was limited to cohort studies developed up to 2014 and evaluated various gestational outcomes in women residing in low- and middle-income countries [12]. Another systematic review on maternal anemia and gestational outcomes published in 2013 was identified; it used both cohort and case-control investigations conducted up to 2010 [13]. In addition, other systematic reviews, published in 2012 and 2015, proposed to evaluate the impact of iron sulfate supplementation on low birth weight [14,15,16]. 

In view of the scarcity of recent review studies regarding the association between maternal anemia and low birth weight that include information from different continents and diverse socioeconomic conditions, this article aimed to systematically assess the relationship between maternal anemia and low birth weight through cohort and case-control studies carried out in several countries around the world. 

## 2. Methods

### 2.1. Registration and Protocol

A search for systematic reviews on the subject was conducted in the International Prospective Register of Systematic Reviews (PROSPERO) database and no records were found. The systematic review was registered in PROSPERO under protocol number CRD42017069451.

### 2.2. Eligibility Criteria for the Studies

The eligibility criteria consisted of cohort and case-control studies assessing the relationship between maternal anemia (hemoglobin levels <11 g/dL) and low birth weight (<2500 g). Investigations involving women diagnosed with anemia of a genetic origin or with self-reported exposure or outcomes were excluded. There was no restriction on the date of publication or the language used.

### 2.3. Information Sources

The search for information was performed up to 5 January 2018. The electronic databases used were Medline, Embase, Scopus, Web of Science, SciELO, and Lilacs. The reference lists of the articles selected for the systematic review were examined to locate citations of references. Additionally, abstracts from congresses and specific databases containing gray literature texts that met the eligibility criteria established in this review were examined.

### 2.4. Search Strategies

The descriptors used and their synonyms were identified in the Medical Subject Headings (MeSH) and Embase Subject Headings (Emtree). The uniterms and Boolean operators in English used in the search strategies were (anemia OR anaemia OR haemoglobin OR hemoglobin OR haematocrit OR hematocrit) AND (Pregnancy OR Pregnant women OR Gravidity OR Maternal exposure OR Mother OR Pregnant OR Gravid OR Obstetric OR Antenatal OR Antepartum OR Gestation) AND (Infant, Low birth weight) AND (Case-control studies OR Retrospective studies OR Case-control study OR Study, Case-control OR Studies, case-control OR Case-comparison studies OR Cohort studies OR Longitudinal studies OR Follow-up studies OR Prospective studies OR Cohort OR longitudinal OR Prospective OR Retrospective OR Incidence study OR Follow-up OR Case control OR Meta-analysis). The search strategy was adapted for the other electronic databases used (Supplementary File 1).

### 2.5. Studies Selection

Two reviewers (ACMGF and PPSP) selected articles by reading titles and abstracts. During the article selection process, the researchers were not aware of the decisions one another made. After this phase, two researchers (ACMGF and RBS) independently read the full text of the previously selected articles. Articles that met the eligibility criteria were included in the systematic review. In cases where there was divergence between the researchers, the inclusion or exclusion of the articles was decided by consensus (ACMGF, PPSP, and RBS). 

### 2.6. Extraction of Data

Data were extracted from the included articles by two independent researchers (ACMGF and RBS) and subsequently confronted. The data were entered into an electronic form in Excel containing the following fields: author’s name, year of publication, place and year of study, objective, study design, sample size, data collection location, data source, criteria for anemia diagnosis, frequency of maternal anemia, percentage of low birth weight infants, association measurements and confidence intervals, and confounding covariables. When the data were not available in the articles, the authors of the studies were contacted. 

### 2.7. Evaluation of Study Quality

The quality of the selected studies was assessed using the Newcastle-Ottawa instrument [17] recommended by the Cochrane Collaboration for cohort and case-control observational studies. It consists of eight questions composed of three axes: study selection, comparability and verification of exposure, and outcome investigated. This instrument has a classification system in which an article receives stars for each criterion met. The categories of quality classification for studies are (1) low quality—when the article receives up to 3 stars, (2) moderate quality—from 4 to 6 stars, and (3) high quality—from 7 to 9 stars. 

### 2.8. Data Analysis 

A statistical description of the studies and the results related to maternal anemia and low birth weight was performed. Statistical heterogeneity was measured using the chi-square test (*p* < 0.10) and the Higgins and Thompson I-square (I^2^), and the magnitude of the inconsistency was evaluated [18]. I^2^ values above 50% were considered high, values of 25% to 50% were considered moderate, and values less than 25% were considered low [18].

The summary of exposure and outcome frequency was calculated with 95% confidence intervals using the Freeman-Tukey double arcsine transformation technique [19]. The DerSimonian-Laird method was used for the random-effects meta-analysis to obtain the global association measurement, odds ratios and 95% confidence intervals [18]. When the cohort study findings were shown to have relative risk, this association measurement was converted to an odds ratio according to criteria defined by Zhang [20]. To evaluate the mean difference in birth weight, the nonstandard technique was used in the meta-analysis of random effects.

Publication bias was analyzed using Begg’s funnel plot and Egger regression with a significance level of 5% [18]. The trim-and-fill test was performed to identify the possible effects of the absence of studies related to the summary association measurement in the meta-analysis. 

Sensitivity analysis, subgroup analyses and meta-regressions were also performed to verify the source of heterogeneity in the studies used in the systematic review. In the subgroup analyses and meta-regressions, the following covariables were used: study design (prospective cohort, retrospective cohort, case-control), data collection location (hospital, primary health units, community), differential diagnosis of maternal anemia (maternal anemia, iron deficiency anemia), hemoglobin levels (<11 g/dL; <10.6 g/dL; <10 g/dL; <8 g/dL), severity levels of maternal anemia as classified in the study (mild, moderate, severe), gestational trimester when maternal anemia was diagnosed (first, second, third), magnitude of the association measurement (<2; ≥2), Human Development Index (very high, high, medium, low), geographic region (America, Africa, Asia, Europe, Oceania), sample size (<1000; ≥1000), and the year the research was initiated (<1990, 1990–2000, 2001–2010, 2011–2017). Data analysis was performed using the statistical package STATA^®^ version 15. (StataCorp LLC, College Station, TX, USA), Serial number: 301506206729.

## 3. Results

### 3.1. Selected Studies

**Figure 1:**
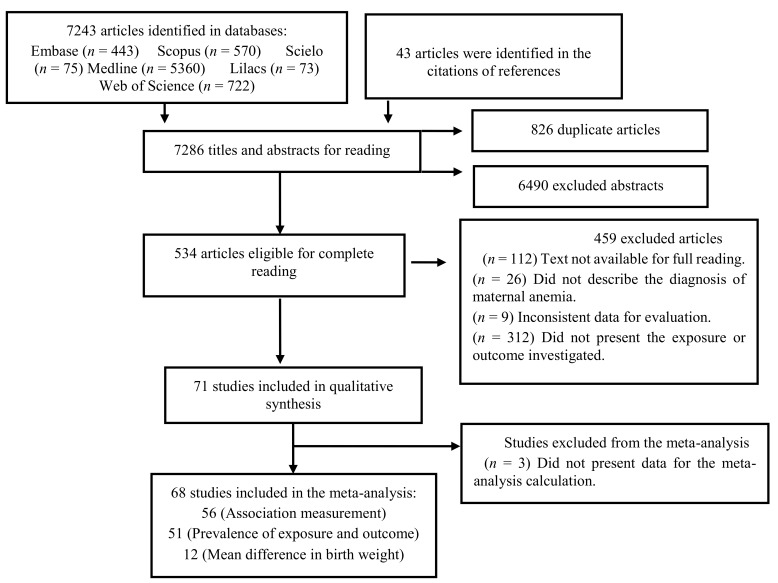
Flowchart of the search, selection, and inclusion of the studies.

From the database searches, 7243 records were identified. After duplicates were removed and titles and abstracts were read, 534 articles were selected for full reading. Only 71 texts met the eligibility criteria of this systematic review (Figure 1). The publication period for the evaluated investigations was from 1986 to 2017.

### 3.2. General Characteristics and Quality of Studies

The population included in this review consisted of 916,990 pregnant women with a mean age of 26 years. Of the total number of selected studies, 54 cohort studies and 17 case-control studies (Table 1) were identified. Much of the research was conducted between 2000 and 2010 in Asian countries with a high Human Development Index, and in a hospital environment.

The definition of maternal anemia used in most studies was hemoglobin levels below 11 g/dL, and more than half of the studies collected this information from medical records. Only 20 studies included information regarding the severity of maternal anemia, and of the six studies that diagnosed pregnant women with iron deficiency anemia, only three reported the ferritin level to confirm the differential diagnosis. The methodological quality of the studies was considered moderate (mean: 6.6), and no selected articles were of low quality. 

For the meta-analysis of maternal anemia proportion, 50 studies were included. To estimate the overall frequency of low birth weight, 51 studies were selected as they contained all the information necessary to generate the summary measurement. Three of the studies included in this review [21,22,23] did not include the association measurement and mean birth weight in their findings. However, the authors stated that maternal anemia was considered a risk factor for low birth weight.

To generate the summary association measurement, 56 studies were included in the meta-analysis as they presented association measurements or information that made the calculation of the odds ratio possible. Of these, 36 presented only crude measurements, 19 presented crude and adjusted measurements, and one investigation included only the adjusted measurement. Among these investigations, the covariables most frequently considered in adjustment of the association measurements were maternal age, maternal level of education, hypertensive gestational disease, and gestational age.

For the summary measurement of the mean difference in birth weight, only 12 studies were selected for the meta-analysis. Although 14 studies measured mean birth weight according to the presence of maternal anemia, two of these were not considered eligible because they did not present sufficient data to calculate the overall measurement [24,25].

### 3.3. Maternal Anemia and Low Birth Weight

The summary frequencies of exposure and outcome, calculated using proportional meta-analysis, were 34% (95% CI: 29%, 40%) and 14% (95% CI: 12%, 15%), respectively.

**Figure 2:**
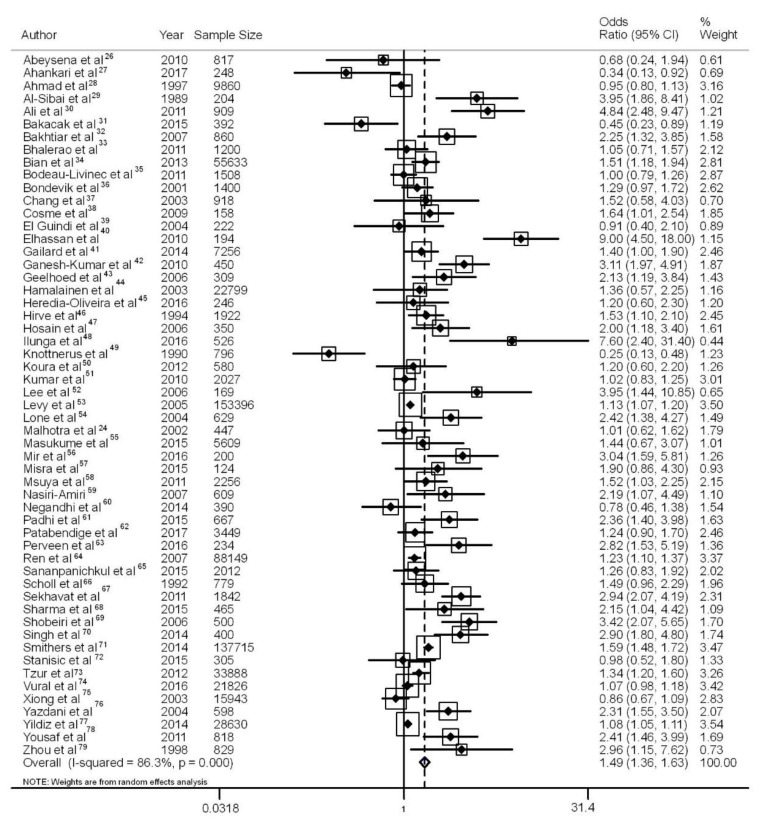
Meta-analysis with crude effect measurement for the evaluated studies and 95% confidence intervals [24,26,27,28,29,30,31,32,33,34,35,36,37,38,39,40,41,42,43,44,45,46,47,48,49,50,51,52,53,54,55,56,57,58,59,60,61,62,63,64,65,66,67,68,69,70,71,72,73,74,75,76,77,78,79].

**Figure 3:**
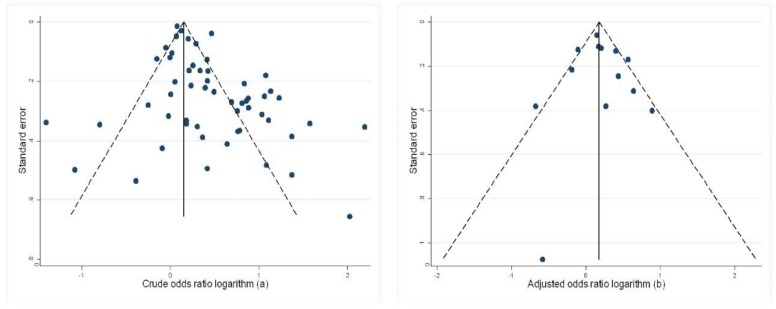
(**a**) Publication bias of the crude odds ratio and (**b**) Publication bias of the adjusted odds ratio.

In this systematic review, the meta-analysis summarized the crude odds ratio as 1.49 (95% CI: 1.36, 1.63) and I^2^ as 86% (Figure 2), which represents a statistically significant association between maternal anemia and low birth weight, although with high heterogeneity. The Egger test (*p* < 0.01) and the funnel plot showed publication bias for the crude association measurement (Figure 3). If there had been no publication bias, the expected association measurement would be 1.18 (95% CI: 1.07, 1.29) according to the trim-and-fill test.

**Figure 4:**
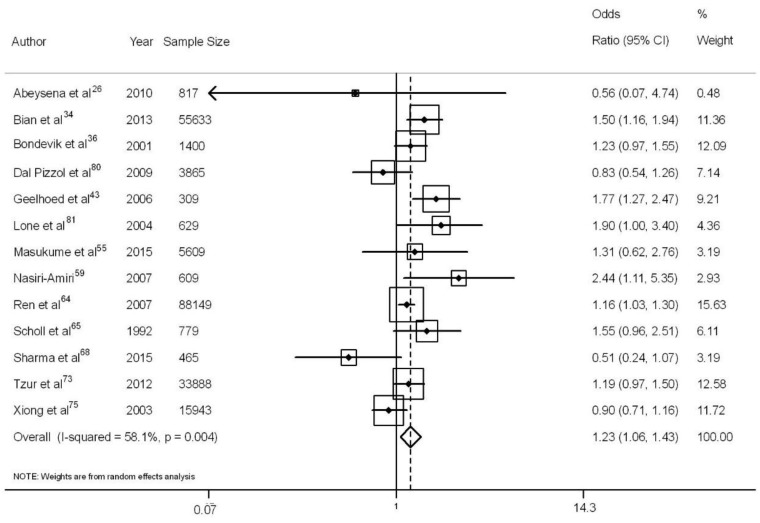
Meta-analysis with adjusted effect measurements for the evaluated studies and 95% confidence intervals [26,34,36,43,55,59,64,65,68,73,75,80,81].

The overall association estimate for the adjusted odds ratio was 1.23 (95% CI: 1.06, 1.43), with 58% heterogeneity (Figure 4) and no publication bias (Egger’s test: *p* = 0.72). This finding was confirmed by the trim-and-fill test, which showed that the number of publications and the association measurement expected consistent with the estimate measured in the present systematic review.

The meta-analysis of global mean difference showed that the children of pregnant women with maternal anemia presented a mean reduction of 60.55 g (95% CI: −111.38, −9.71, I^2^: 96%) in birth weight compared with the children of women with normal hemoglobin levels, and there was no publication bias (Egger: *p* = 0.611). The trim-and-fill test confirmed that the findings presented are similar to the expected mean difference.

The sensitivity analysis identified 9 studies that were considered outliers and that produced distortion in the crude [83,84,85] and adjusted [24,40,42,48,72,83,86] summary measurements, although they were included in the qualitative evaluation of the investigations. The subgroup analysis for the crude and adjusted odds ratio indicated that for most of the variables evaluated, the association measurement continued to be associated with low birth weight, even after stratification (Table 2). Multiple meta-regressions for the crude association measurement confirmed that the strength of the association measurement (*p* < 0.01), geographic location (*p* = 0.02), and sample size (*p* < 0.01) may have been the possible causes of heterogeneity. For the adjusted association measurement, the sample size (*p* = 0.01) and the methodological quality of the studies (*p* = 0.01) were the possible explanation.

## 4. Discussion

The main findings of this systematic review show that maternal anemia is a risk factor for low birth weight. These results were confirmed through a meta-analysis of the mean difference in birth weight, which showed that the children of women with maternal anemia had a reduction in birth weight compared with those whose mothers did not develop anemia. The methodological quality of the longitudinal studies used in this systematic review, which included case-control and cohort designs, was considered moderate to high, and the studies were performed in different countries across all continents.

Of the previous systematic reviews on the subject, four corroborated the present findings. They also showed moderate and high heterogeneity in their meta-analyses; however, they only used longitudinal studies to evaluate the relationship between maternal anemia and neonatal events and did not focus specifically on low birth weight [12,13,87,88]. Rahman et al. [12] evaluated exposure and outcomes in 17 cohort studies with high methodological quality, but only included data from low- and middle-income countries. Sukrat et al. [13] used only two databases to track their studies on this topic, and to perform the meta-analysis, they included association measurements from only 10 longitudinal studies according to a cutoff point based on hemoglobin levels.

The other two reviews [87,88] included cross-sectional investigations in addition to cohort and case-control studies. This fact may have contributed negatively to the results of their meta-analyses since the temporality of the events was not considered in the estimation of the summary association measurement. Only Anand and Leonardi-Bee [87] corroborated the present review’s findings from the meta-analysis of the mean difference in birth weight; however, they used a small sample size of only five studies.

This systematic review also provided an estimation of the global frequency of maternal anemia originating from the proportional meta-analysis, which corroborates the most recent official data [8]. Although there is significant regional variation in the occurrence of maternal anemia, the summary measurement presented in this systematic review was able to reproduce this proportion more reliably at the global level because it included studies from countries on all continents, regardless of their socioeconomic conditions.

The proportion of studies conducted in Asian countries was much higher than in other countries on other continents and it could have influenced association measurements in the subgroup analysis according to geographic region. On the other hand, if the findings were evaluated through the Human Development Index (HDI), the measurement indicates more clearly the presence of an association between maternal anemia and low birth weight, in the different countries according to the socioeconomic stratum of the referred populations. Similarly, the overall percentage of low birth weight children is similar to that reported by the World Health Organization for various countries [89].

Most of the studies in this systematic review presented findings based on a diagnosis of maternal anemia that used a lower severity cutoff. This fact shows how a narrow diagnosis of maternal anemia predominates in the studies. In the present systematic review, the positive association between maternal anemia and low birth weight was verified using subgroup analyses that included both the presence of anemia and its severity level due to the need for more accurate anemia diagnosis.

Another important issue is the high heterogeneity found among the studies, which could be attributed to regional differences in research; to specific characteristics of populations, such as health, socioeconomic and nutritional status; or to the different diagnostic definitions of maternal anemia [6,90]. Countries with a low Human Development Index show an increase in the magnitude of the association between maternal anemia and low birth weight; this association is supported by the findings of this review and justifies the high heterogeneity encountered in the present study.

Of the variables mentioned above, only the Human Development Index was identified as a source of heterogeneity in the subgroup analysis of the present systematic review. Multiple meta-regression showed that the high I^2^ in the crude association measurement could be partially explained by geographic region, sample size. and the strength of the association measurement of the studies used in the meta-analysis. For the adjusted measurement, the sample size and methodological quality of the studies were possible explanations for the high heterogeneity observed.

Regarding the limitations of this review, the publication bias of the crude association measurement stands out, despite a wide search in several databases that had not been previously investigated in other systematic reviews. In an attempt to minimize bias, texts from the gray literature were inserted, and the authors of the published articles that did not provide some data considered relevant for this review were contacted.

Furthermore, in relation to prematurity, although this is strongly associated with low birth weight, it was not included in this meta-analysis due to the scarcity of information about gestational age related to birth weight and maternal anemia. Consequently, the global association measurements between the exposure and the outcome, by gestational age subgroups, could not be obtained in this investigation.

To identify the possible effects of the absence of studies from the meta-analysis of the summary association measurement, the trim-and-fill test for the crude association measurement was used. As a result, a 19% reduction in the odds ratio was observed, together with the need for an additional 14 articles to be added to the meta-analysis to correct this measurement. In the present review, 71 studies were included, but only 58 presented sufficient information to calculate the crude summary measurement of the meta-analysis, demonstrating that 13 studies may have shown reporting bias. Of these, three were excluded because they were considered outliers and may have led to an overestimation of the meta-analytic odds ratio. However, when the publication bias was evaluated with and without the inclusion of these outliers, no change in the results was observed. 

The difference between the crude and adjusted summary association measurement was lower than 17%, showing that although there one of the measurements showed publication bias, the studies’ findings point in the same direction: namely, they indicate that maternal anemia may be an important risk factor for low birth weight. However, this result should be interpreted with caution due to the high heterogeneity among the studies, which can be considered an indicator of inconsistency [91,92,93,94,95,96].

Another limitation was the use of different risk measurements (relative risk and odds ratios) in the meta-analysis. To correct the possible effects of this combination of measurements, all relative risks were converted into odds ratios. To verify the effect of this conversion on the meta-analysis, a sensitivity analysis was performed, and the articles that presented a relative risk and an outcome occurrence higher than 10% were excluded [20]. However, the difference was insignificant. Another aspect that reinforces this finding is that the two types of association measurements have a tendency to present similar results since the outcome investigated is considered rare, which minimizes the possible impact of this measurement difference [20].

The strengths of this review include the high number of databases employed, the use of research techniques and validated instruments, such as the Newcastle Quality Survey Scale-Ottawa [17] and the Meta-analysis of observational studies in epidemiology (MOOSE), to evaluate the studies and draft the systematic reviews [97]. In addition, the subgroup analyses to measure possible associations between exposure and outcome used covariables that are considered epidemiologically important.

In terms of minimizing the information bias, another positive aspect was the use of original surveys that obtained information from reliable exposure and outcome measures, such as laboratory tests and medical records, rather than self-reported data. 

To the best of our knowledge, this is the first systematic review to include research on the subject conducted in all continents using a large number of longitudinal studies. Although the present findings indicate that maternal anemia is associated with low birth weight, they also signal the need for further longitudinal research on this topic, with an evaluation of the various types of anemia in pregnant women. The relationship between anemia and low birth weight could be better understood through more robust methods, which would improve the quality of scientific evidence on the topic and provide more effective preventive and health-promotion measures for mothers and children.

## Figures and Tables

**Table 1:** Study characteristics.

Characteristic	*N*	%
**Study design**		
Prospective cohort	38	53.6
Retrospective cohort	16	22.5
Case-control	17	23.9
**Location of data collection**		
Hospital	58	81.7
Primary health units	7	9.9
Community	3	8.4
**Differential diagnosis of exposure**		
Maternal anemia	65	91.6
Iron deficiency anemia	6	8.4
Hemoglobin levels *		
<11 g/dL	54	77.1
<10 g/dL	10	14.3
<8 g/dL	6	8.6
**Geographic region**		
America	9	12.7
Africa	6	8.4
Asia	44	62.0
Europe	8	11.3
Oceania	4	5.6
**Sample size**		
≤1000	43	60.6
>1000	28	39.4
**Methodological quality of the studies**		
Moderate	40	56.3
High	31	43.7
Year the research was initiated **		
Before 1990	8	14.0
1990–2009	12	21.0
2000–2009	25	44.0
2010–2017	12	21.0
**Control for confounding**		
Yes	20	28.2
No	51	71.8

* One study did not present the definition but affirmed that there was a reduction in birth weight. ** Some studies did not report the start date of the survey.

**Table 2:** Subgroup analysis and meta-regression of the crude and adjusted effect measurement.

Variable	*N*	Crude OR (95% CI)	Heterogeneity (I^2^)	Meta-Regression *p* Value *	*N*	Adjusted OR (95% CI)	Heterogeneity (I^2^)	Meta-Regression *p* Value *
**Study design**								
Prospective cohort	30	1.51 (1.29–1.76)	83.8%		6	1.38 (0.96–2.01)	46.7%	
Retrospective cohort	13	1.18 (1.08–1.28)	72.7%	0.03	5	1.24 (1.04–1.49)	70.8%	0.56
Case-control	12	2.29 (1.51–3.47)	82.8%		2	0.85 (0.37–2.00)	79.4%	
**Data collection location**								
Hospital	44	1.54 (1.39–1.71)	85.4%		10	1.30 (1.06–1.59)	63.4%	
Primary health units	6	1.28 (1.02–1.61)	68.9%	0.28	2	1.05 (0.77–1.42)	55.2%	0.50
Community	5	1.40 (1.09–1.78)	60.4%		1	-	-	
**Differential diagnosis of exposure**								
Maternal anemia	51	1.47 (1.34–1.61)	86.8%	0.54	12	1.21 (1.04–1.42)	60.0%	0.53
Iron deficiency anemia	4	1.89 (1.10–3.27)	60.9%		1	-	-	
**Hemoglobin levels (g/dL)**								
<11	33	1.48 (1.32–1.66)	86.7%		10	1.25 (1.04–1.49)	50.3%	
<10.6	5	2.58 (1.69–3.94)	72.4%	0.87	1	-	-	0.84
<10	7	1.25 (1.02–1.52)	86.2%		2	1.04 (0.79–1.37)	64.0%	
<8	3	1.72 (1.10–2.69)	33.6%		0	-	-	
**Severity levels of maternal anemia**								
Mild	7	1.14 (0.99–1.31)	66.3%		0	-	-	
Moderate	9	1.39 (1.11–1.74)	49.0%	0.61	2	1.16 (1.04–1.29)	0.0%	-
Severe	9	2.31 (1.47–3.63)	82.7%		4	2.24 (1.35–3.71)	0.0%	
**Odds ratio**								
<2	34	1.15 (1.06–1.25)	80.1%	<0.01	10	1.14 (0.99–1.30)	47.8%	0.02
≥2	21	2.85 (2.48–3.27)	16.0%		3	1.87 (1.42–2.45)	0.0%	
**Maternal anemia by gestational trimester**								
First	3	1.51 (0.94–2.42)	55.7%		0	-	-	
Second	3	0.98 (0.56–1.68)	61.5%	-	0	-	-	-
Third	3	0.88 (0.53–1.48)	72.2%		0	-	-	
Human Development Index **								
Very high	12	1.24 (1.03–1.49)	88.4%		3	1.25 (1.03–1.51)	0.0%	
High	15	1.30 (1.14–1.49)	82.0%	0.02	6	1.15 (0.92–1.44)	64.8%	0.92
Medium	21	1.82 (1.46–2.27)	77.3%		4	1.30 (0.87–1.94)	72.4%	
Low	7	2.28 (1.23–4.21)	89.5%		1	-	-	
**Geographic region**								
America	4	1.38 (1.00–1.91)	0.0%		1	-	-	
Africa	6	3.07 (1.60–5.89)	83.9%		1	-	-	0.76
Asia	38	1.44 (1.31–1.58)	83.7%	0.30	9	1.20 (1.02–1.40)	58.2%	
Europe	4	0.94 (0.47–1.89)	87.4%		1	-	-	
Oceania	3	1.51 (1.23–1.85)	16.0%		1	-	-	
**Sample size**								
≤1000	34	1.84 (1.46–2.33)	79.0%	0.02	6	1.46 (1.00–2.16)	57.2%	0.15
>1000	21	1.24 (1.13–1.35)	86.8%		7	1.15 (1.02–1.31)	44.1%	
**Year the research was initiated**								
Before 1990	4	0.96 (0.58–1.60)	91.5%		1	-	-	
1990–1999	9	1.34 (1.10–1.64)	81.1%	0.36	4	1.22 (1.00–1.50)	64.6%	0.08
2000–2009	22	1.75 (1.44–2.13)	82.3%		6	1.40 (1.16–1.69)	16.0%	
2010–2017	11	1.32 (1.09–1.60)	87.2%		0	-	-	
**Methodological quality of the studies**								
Moderate	30	1.65 (1.42–1.91)	83.2%	0.25	2	0.52 (0.26–1.04)	0.0%	0.05
High	25	1.49 (1.36–1.63)	87.0%		13	1.27 (1.10–1.46)	56.6%	

* *p* value < 0.05. The *p* value represents the difference between the characteristics; ** Human Development Report, 2016 [82].

## Supplementary Materials

The following are available online at http://www.mdpi.com/2072-6643/10/5/601/s1, Supplementary 1: Search strategy.
