# Supplementary material for: Maternal Anemia and Low Birth Weight: A Systematic Review and Meta-Analysis
Source: Nutrients. 2018 May 12;10(5):601. doi: 10.3390/nu10050601 (PMC5986481; doi:10.3390/nu10050601)
Supplement: Supplementary file 1 [file nutrients-10-00601-s001.pdf]

Supplementary File

Supplementary 1. Search strategy

Medline (pubmed) n= 5360	(“anemia” [Title/Abstract] OR "anemia"[MeSH Terms] OR “anaemia” [Title/Abstract] OR “anaemia” [MeSH Terms] OR “haemoglobin” [Title/Abstract] OR “haemoglobin” [MeSH Terms] OR “hemoglobin” [Title/Abstract] OR “hemoglobin” [MeSH Terms] OR “haematocrit” [Title/Abstract] OR “hematocrit” [Title/Abstract] OR “haematocrit” [MeSH Terms] OR “hematocrit” [MeSH Terms]) AND (“Pregnancy” [MeSH Terms] OR “Pregnancy” [Title/Abstract] OR “Pregnant Women” [Title/Abstract])] OR “Pregnant Women” [MeSH Terms] OR “gravidity” [Title/Abstract])] OR “gravidity” [MeSH Terms] OR “maternal exposure” [MeSH Terms] OR “maternal exposure” [Title/Abstract] OR “mother” [Title/Abstract] OR “mother” [MeSH Terms] OR “pregnan” [Title/Abstract] OR “pregnan” [MeSH Terms] OR “gravid” [Title/Abstract] OR “gravid” [MeSH Terms] OR “obstetric” [Title/Abstract] OR “obstetric” [MeSH Terms] OR “antenatal” [Title/Abstract] OR “antenatal” [MeSH Terms] OR “antepartum” [Title/Abstract] OR “antepartum” [MeSH Terms] OR “gestation” [Title/Abstract] OR “gestation” [MeSH Terms]) AND (“Infant, Low Birth Weight” [MeSH Terms] OR “Infant, Low Birth Weight” [Title/Abstract] OR “Low Birth Weight” [Title/Abstract]) AND(“case-control studies” [MeSH Terms] OR “case-control studies” [Title/Abstract] OR “retrospective studies” [Mesh] OR “retrospective studies” [Title/Abstract] OR “case-control study” [Title/Abstract] OR “Study, case-control” [Title/Abstract] OR “Studies, case-control” [Title/Abstract] OR “case-comparison studies” [Title/Abstract] OR “cohort studies” [MeSH Terms] OR “cohort studies” [Title/Abstract] OR “Longitudinal Studies” [MeSH Terms] OR “Follow-Up Studies” [MeSH Terms] OR “Prospective studies” [MeSH Terms] OR “cohort” [Title/Abstract] OR “longitudinal” [Title/Abstract] OR “prospective” [Title/Abstract] OR “retrospective” [Title/Abstract] OR “incidence study” [Title/Abstract] OR “follow up” [Title/Abstract] OR “case control” [Title/Abstract] OR “meta-analysis” [MeSH term])
Scopus n= 570	(TITLE-ABS-KEY(("anemia" OR “anaemia” OR “haemoglobin” OR “hemoglobin” OR “haematocrit” OR “hematocrit”)) AND TITLE-ABS-KEY (("Pregnancy" OR "Pregnant women” OR “gravidity” OR “maternal exposure" OR “Gestation” OR “Prenatal Care” OR “mother” OR “pregnan” OR “gravid” OR “obstetric” OR “antenatal” OR “antepartum” OR “gestation”)) AND TITLE-ABS-KEY (("Infant, Low Birth Weight" OR "Low Birth Weight" OR Low-Birth-Weight Infant)) AND TITLE-ABS-KEY (("retrospective studies" OR "case-control studies” OR “case-comparison studies" OR "cohort studies" OR "prospective studies" OR "longitudinal studies" OR "Incidence studies" OR "Incidence Study" OR “Retrospective Study” OR “Longitudinal Survey” OR “Analysis, Cohort” OR “meta-analysis”)))
Embase (sem medline) n= 443	'anemia'/exp OR 'anemia' OR 'anaemia'/exp OR 'anaemia' OR 'haemoglobin'/exp OR 'haemoglobin' OR 'hemoglobin'/exp OR 'hemoglobin' OR 'haematocrit'/exp OR 'haematocrit' OR 'hematocrit'/exp OR 'hematocrit' AND ('pregnancy'/exp OR 'pregnancy' OR 'pregnant women'/exp OR 'pregnant women' OR 'gravidity'/exp OR 'gravidity' OR 'maternal exposure'/exp OR 'maternal exposure' OR 'environmental exposure'/exp OR 'environmental exposure' OR 'mother'/exp OR 'mother' OR 'pregnan' OR 'gravid' OR 'obstetric' OR 'antenatal' OR 'prenatal care'/exp OR 'prenatal care' OR 'antepartum' OR 'gestation'/exp OR 'gestation') AND ('low birth weight'/exp OR 'low birth weight') AND ('case-control studies'/exp OR 'case-control studies' OR 'retrospective studies'/exp OR 'retrospective

	studies' OR 'case-control study'/exp OR 'case-control study' OR 'study, case-control' OR 'studies, case-control' OR 'case-comparison studies' OR 'cohort studies'/exp OR 'cohort studies' OR 'prospective studies'/exp OR 'prospective studies' OR 'longitudinal studies'/exp OR 'longitudinal studies' OR 'incidence studies' OR 'studies, incidence' OR 'study, incidence' OR 'cohort study'/exp OR 'cohort study' OR 'cohort analysis'/exp OR 'cohort analysis' OR 'longitudinal study'/exp OR 'longitudinal study' OR 'prospective study'/exp OR 'prospective study' OR 'case control study'/exp OR 'case control study' OR 'retrospective study'/exp OR 'retrospective study' OR 'cohort' OR 'longitudinal'/exp OR 'longitudinal' OR 'prospective' OR 'retrospective' OR 'incidence study' OR 'case' OR 'meta-analysis'/exp OR 'meta-analysis') NOT [medline]/lim
Web of science<br>n= 722	(anemia OR anaemia OR haemoglobin OR hemoglobin OR haematocrit OR hematocrit) AND (Pregnancy OR Pregnant Women OR gravidity OR mother OR pregnan OR gravid OR obstetric OR antenatal OR antepartum OR gestation) AND (Infant, Low Birth Weight OR Low Birth Weight) AND (retrospective studies OR retrospective study OR case-control studies OR case-control study OR case-comparison studies OR case-comparison study OR cohort studies OR cohort study OR prospective studies OR prospective study OR longitudinal studies OR longitudinal study OR Incidence studies OR Incidence Study OR Follow-Up Studies OR cohort OR longitudinal OR prospective OR retrospective OR incidence study OR follow up OR case control OR meta-analysis)
Lilacs<br>n = 73	(Low birth weight) AND (anemia in pregnancy)
SciELO<br>n= 75	(Low birth weight) AND (anemia in pregnancy)
